# EFFECTS OF SPINELESS ON SURVIVAL AND REPRODUCTION IN DROSOPHILA MELANOGASTER

**DOI:** 10.1093/geroni/igae098.3656

**Published:** 2024-12-31

**Authors:** Shelton Swint, Jessica Hoffman

**Affiliations:** Augusta University, Augusta, Georgia, United States; Augusta University, Augusta, Georgia, United States

## Abstract

The aryl hydrocarbon receptor (AhR) is a widely conserved, ligand-activated mammalian transcription factor involved in numerous physiological processes, including maintaining stem cell pluripotency during development and regulating xenobiotic detoxification. One group of commonly studied activators of AhR are tryptophan and its downstream metabolites. Recent studies have shown that AhR-deficient mice display similar survival trends to control animals during early life (≤12 months) and then display a premature aging phenotype. As mice live for two to three years, we can advance our knowledge of AhR and aging using the fruit fly (D. melanogaster) as an alternative. Flies possess an ortholog of the mammalian AhR called spineless (ss), but the effects of ss on survival have yet to be shown. Additionally, many tryptophan-derived metabolites, like tryptamine, have been shown to decrease lifespan in control flies, potentially due to activation of AhR. We found that ss-deficient flies are shorter-lived than wildtype flies, and similar to trajectories in mice, ss flies have normal survival until around 40 days of age. The lifespan-shortening effects of tryptophan metabolites were reduced in ss-deficient flies, suggesting that the negative effects of tryptophan metabolites may be dependent on ss. Furthermore, oxidative stress assays showed that ss flies are more stress resistant, significantly outliving the wildtype flies at an early age. The effects of ss are also being investigated using a potential AhR-antagonist, cis-Jasmone. Overall, our work demonstrates that ss-deficient flies have reduced lifespan and altered responses to tryptophan metabolites, suggesting a conserved role of AhR in the aging process.

